# Recent Advances in the Fabrication and Application of Screen-Printed Electrochemical (Bio)Sensors Based on Carbon Materials for Biomedical, Agri-Food and Environmental Analyses

**DOI:** 10.3390/bios6040050

**Published:** 2016-09-28

**Authors:** Gareth Hughes, Kelly Westmacott, Kevin C. Honeychurch, Adrian Crew, Roy M. Pemberton, John P. Hart

**Affiliations:** Centre for Research in Biosciences, Faculty of Health and Applied Sciences, University of the West of England, Bristol, Coldharbour Lane, Bristol BS16 1QY, UK; gareth6.hughes@uwe.ac.uk (G.H.); Kelly2.Westmacott@live.uwe.ac.uk (K.W.); Kevin.Honeychurch@uwe.ac.uk (K.C.H.); adrian.crew@uwe.ac.uk (A.C.); roy.pemberton@uwe.ac.uk (R.M.P.)

**Keywords:** biosensor, amperometry, voltammetry, screen-printed, biomedical, agri-food, Environmental

## Abstract

This review describes recent advances in the fabrication of electrochemical (bio)sensors based on screen-printing technology involving carbon materials and their application in biomedical, agri-food and environmental analyses. It will focus on the various strategies employed in the fabrication of screen-printed (bio)sensors, together with their performance characteristics; the application of these devices for the measurement of selected naturally occurring biomolecules, environmental pollutants and toxins will be discussed.

## 1. Introduction

This review explores the fabrication of analytical devices applied to analytical challenges within the biomedical, agri-food and environmental sectors. These electrochemical (bio)sensors use screen-printed carbon electrodes (SPCEs) which are often modified to enhance sensitivity and selectivity. Carbon has many structural forms and each possess different chemical properties; these can be selected to enhance an electrochemical response making carbon a favourable electrode material. Alone, screen-printed carbon can act as a sensitive electrode material however it is often utilized as a base platform to which modifications can be applied. The recent advances in modifying materials and deposition techniques will be discussed in the following sections. From the early 1990s, screen-printed devices increased in popularity and subsequent advances have been reviewed periodically [1,2,3,4,5,6,7], the publications discussed herein will encompass the developments, over the past decade, of (bio)sensors based on SPCEs.

The review is broadly divided into four sections based on the classes of analyte determined, these are: naturally occurring biomarkers, some water soluble vitamins, organophophate pesticides and metal ions.

## 2. Screen-Printed Carbon Based Biosensors for the Determination of Glucose, Galactose, Glutamate, Lactate and Proteins

### 2.1. Glucose

In this section, the fabrication methods are discussed according to the technique of enzyme immobilization. Important analytical properties such sensitivity, linear range and the method of immobilization have been summarized in Table 1. The amperometric response generated by the glucose biosensors, in the presence of glucose oxidase and a mediator, can generally be described by the following reactions [7]:

Glucose + GOD_OX_ → Gluconolactone + GOD_RED_

GOD_RED_ + Mediator_OX_ → GOD_OX_ + Mediator_RED_

Mediator_RED_ → Mediator_OX_ + ne^−^

The method of adsorption makes use of physical interactions between a binding agent and the enzyme. These interactions include van der Waals forces, ionic interactions and hydrogen bonding. These interactions are typically relatively weak compared to other immobilisation strategies, however they do not compromise the structure of the enzyme active sites, which allows the enzyme to retain its activity. Examples of adsorptive enzyme carriers which have been used in conjunction with SPCEs include, chitin, chitosan, silica, polyurethane and poly(oxyethylene glycol).

Piermarini et al. [8] have reported a glucose biosensor for the monitoring of micro-alcoholic fermentations in red wine. GOD was immobilised onto a screen-printed electrode with glutaraldehyde and Nafion. The amperometric responses for the biosensor are not shown; as a result the sensitivity of the biosensor cannot be deduced. A recovery study in diluted red wine has shown excellent recovery with a precision of <5%.

Measuring glucose subcutaneously through the skin is of great scientific interest. Jiang et al. [9] have developed a glucose biosensor by drop-coating an Os-complex mediator and a solution of GOD, glutaraldehyde and BSA onto the surface of a thin film gold electrode. The biosensor was successfully applied to the determination of glucose extracted from the skin by reverse iontophoresis. The biosensor demonstrated a proportional amperometric response to increasing subcutaneous glucose levels. The linear range compares favorably with that reported by Piermarini et al. [8].

A device for measuring glucose in honey and blood using a simple fabrication technique was reported [10]. The immobilization procedure consisted of drop-coating a mixture of horseradish peroxidase (HRP) and GOD onto the surface of a screen-printed ferrocyanide/carbon electrode. The sensitivity (−2.12 µA/mM) improves upon previously discussed biosensors [8,9]. The biosensor was successfully applied to real samples and demonstrated excellent glucose recoveries with high reproducibility.

Entrapment is defined as the integration of an enzyme with a polymer matrix, whilst retaining the structure of the enzyme. The entrapment matrix can also serve as a barrier to interfering species which may be present in complex media such as serum and food.

A reagentless glucose biosensor developed by Gao et al. [11] was successfully applied to both amperometric and flow injection studies. The biosensor was constructed by electrodepositing alternating layers of GOx-SWCNTs and PVI-Os until a multi-layer structure is formed. An interference study demonstrated large currents in response to both uric acid and ascorbic acid. However, the biosensor possesses the highest sensitivity (32 μA·mM^−1^·cm^−2^) in comparison to other biosensors constructed by the adsorption of the enzymes onto the surface of SPCEs.

In a subsequent short communication, Gao et al. [12] have reported a significant reduction in the currents generated by the interferences by the addition of a Nafion membrane. As expected, the addition of the Nafion layer has resulted in a reduction of the biosensor sensor sensitivity (from 32 μA·mM^−1^·cm^−2^ to 16.4 μA·mM^−1^·cm^−2^) and an increase in the linear range (from 500–800 μM to 200–6000 μM). The sensitivity improves upon the sensitivity reported by Jiang et al. (28.24 nA·mM^−1^·cm^−2^) [9].

**Figure 1:**
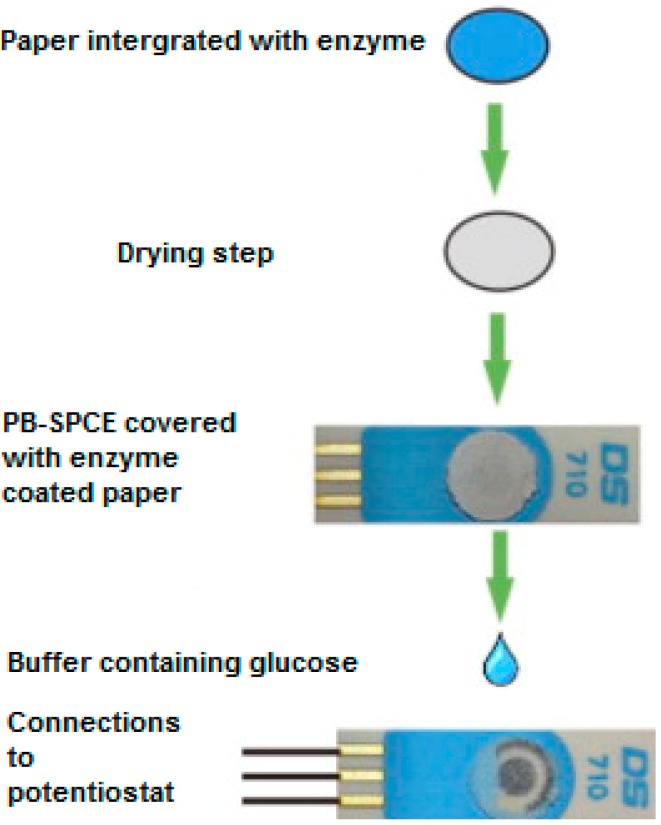
Schematic illustration of glucose oxidase paper disc preparation and integration with the prussian blue-screen-printed carbon electrodes (SPCE). Adapted from [13].

Using a very different strategy, Sekar et al. [13] described a novel method of immobilizing glucose oxidase to the surface of a Prussian Blue modified screen-printed electrode. The enzyme was drop-coated onto a disk of porous cellulose paper which, once dry, was securely fixed to the surface of a PB-SPCE (Figure 1). As a result, the analysis of glucose was carried out by dropping glucose solutions directly onto the surface of the paper disc. The biosensor possesses excellent cost-effectiveness and was constructed using a very simple fabrication technique.

The integration of glucose oxidase into a screen-printed carbon electrode is particularly suited to the mass production of low-cost disposable glucose biosensors. The enzyme has proved sufficiently stable to be encapsulated within a water-based carbon ink without altering the conformational structure of the enzyme. Pemberton et al. [14] applied this method to the development of microband glucose biosensors for the determination of glucose in serum, the results of which compared favorably with a standard spectrophotometric assay. The microband biosensor was successfully applied to the monitoring of glucose metabolism by human hepatocyte carcinoma cells (HepG2) [15] and subsequently developed and applied to the real time monitoring of cellular toxicity [16,17].

In a more complex fabrication processes, Chiu et al. [18] immobilised glucose oxidase onto the surface of a screen-printed carbon electrode by sequential electrodeposition. The enzyme was entrapped onto the surface of a screen-printed carbon electrode by the sequential electrodeposition of poly(3,4-ethylenedioxythiophene), Prussian Blue and multi-walled carbon nanotubes. An extensive linear range of 1 to 10 mM was reported.

A disposable glucose oxidase biosensor was developed by Zuo et al. [19] for the determination of blood glucose in a rabbit serum sample. The enzyme was encapsulated by a mixture of silica sol-gel and PVA, combined with a colloidal silver nanoparticles solution. The mixture was then sonicated for one minute and drop-coated onto the surface of the screen-printed electrode. The biosensor possesses a linear range that extends over two orders of magnitude and a higher sensitivity (20.09 mA·M^−1^·cm^−2^) than previously described biosensors.

### 2.2. Galactose

For the following reports on galactose determination, the performance characteristics are summarized in Table 2. A disposable amperometric biosensor for the measurement of galactose has been described by Kanyong et al. [20]. The fabrication process consists of drop-coating 1% cellulose acetate (CA) onto the surface of a cobalt phthalocyanine screen-printed carbon electrode (CoPC-SPCE). Once dry, an aliquot of galactose oxidase was drop-coated onto the surface of the CA-CoPC-SPCE and left to air-dry. The biosensor has been applied to the determination of galactose in fortified and unfortified bovine serum. A mean recovery value of 99.9% (*n* = 6) was attained, with a low coefficient of variation of 1.10%, implying a high level of reproducibility.

In a subsequent report, a microband galactose biosensor [21] was applied to the determination of galactose taken up by hepatocellular carcinoma cells (HepG2). In order to measure the toxicity of paracetamol to HepG2 cells, the cells were incubated with 10 mM galactose and different concentrations of paracetamol for 24 h. The enzyme was immobilised utilizing a similar method. The microband biosensor demonstrated greater sensitivity (7.27 µA·mM^−1^·cm^−2^) to galactose in comparison to a conventionally sized biosensor (7.00 µA·mM^−1^·cm^−2^).

### 2.3. Glutamate

**Figure 2:**
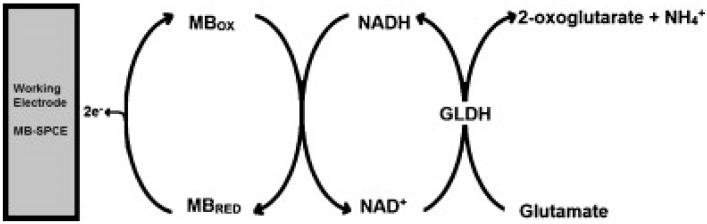
Schematic displaying the interaction between the immobilized enzyme GLDH and glutamate at the surface of the electrode and the subsequent generation of the analytical response. Reproduced with permission from [22].

For the following reports on glutamate determination, the performance characteristics are summarized in Table 3. Hughes et al. [22] have described the fabrication of an amperometric screen-printed glutamate biosensor based on the enzyme glutamate dehydrogenase (GLDH). GLDH was immobilised to the surface of a Meldola’s Blue screen-printed biosensor (MB-SPCE) by chitosan (CHIT). CHIT is a linear polysaccharide which possesses excellent film-forming properties and is commonly used as an immobilization matrix for enzymes [23]. The biosensor was successfully applied to the determination of glutamate in food and clinical samples. The reaction scheme which leads to the production of the analytical signal is shown in Figure 2.

**Figure 3:**
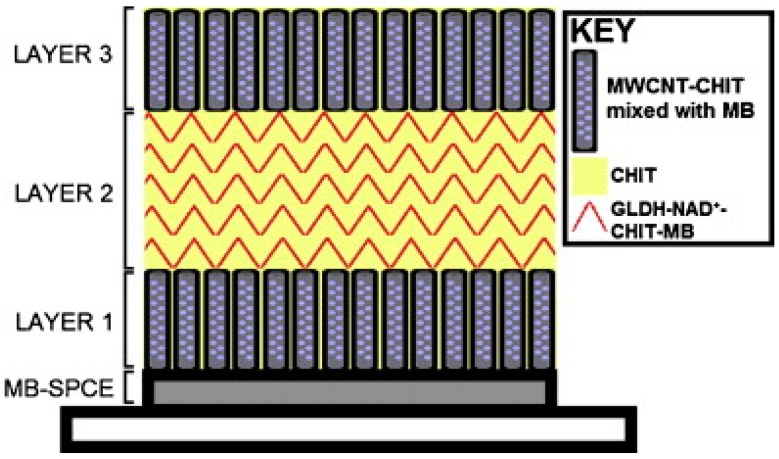
A schematic diagram displaying the layer-by-layer drop coating fabrication procedure used to construct the reagentless glutamate biosensor, based on a MB-SPCE electrode. Reproduced with permission from [24].

The biosensor was further developed by immobilizing all the components onto the surface of the transducer [24]. A layer-by-layer drop-coating fabrication procedure was employed, as illustrated in Figure 3. The enzyme and cofactor were immobilised in a mixture of CHIT and multi-walled carbon nanotubes on the surface of a MB-SPCE. The biosensor response compared favourably to the previously discussed glutamate biosensor [22], where NAD^+^ was present in free solution.

Khan et al. [25] have described the detection of nanomolar concentrations of glutamate by utilizing screen-printed electrodes modified with carbon nanotubes. The biosensors were prepared by drop-coating glutamate oxidase onto the surface of a carbon nanotube modified SPCE and left to dry overnight. The biosensor possesses a detection limit of 10 nM. It is worth noting that all the experiments were carried out at room temperature; as a result, it would be of interest to see if the sensitivity of the biosensor could be increased further by increasing the temperature to the optimum temperature of the enzyme.

### 2.4. Lactate

For the following reports on biosensors for lactate determination, performance characteristics are summarized in Table 4. Radoi et al. [26] have described the development of an amperometric biosensor for the determination of lactic acid in probiotic yoghurts. The biosensor was fabricated by drop-coating a solution of lactate dehydrogenase mixed with neutralized Nafion, onto the surface of a variamine blue modified screen-printed electrode (VB-SPE).

A similar method was adopted by Piano et al. [27]. The lactate biosensor was fabricated by drop-coating lactate dehydrogenase and NAD^+^ onto the surface of a screen-printed carbon electrode containing a Meldola’s Blue-Reinecke Salt mediator. The enzyme and cofactor were immobilised by drop-coating a layer of cellulose acetate on top of the device. The cellulose acetate acts as a perm-selective membrane, preventing the cofactor and enzyme leaching into solution. This led to an increase in the sensitivity and the linear range of the biosensor in comparison the biosensor reported by Radoi et al. [26].

A microband lactate biosensor was fabricated by integrating lactate oxidase directly into the water-based ink formulation, printing, then subsequently cutting this to form a microband [28]. By integrating the enzyme within a water-based ink, the enzyme does not leech into free solution, but retains its conformational structure. The micro-biosensor was successfully applied to the determination of lactate in phosphate buffer saline (PBS) in unstirred solutions, suggesting that the biosensor is appropriate for further studies in cell culture media.

In addition to Nafion, glutaraldehyde is commonly used as an enzyme immobilization agent. Pereira et al. [29] have described the fabrication of a lactate biosensor by immobilizing lactate dehydrogenase and NAD^+^ utilizing a mixture of multi-walled carbon nanotubes, glutaraldehyde and bovine serum albumin. The mediator, Meldola’s Blue (MB), was adsorbed to the multi-walled carbon nanotubes, resulting in high surface area and conductivity. The biosensor was successfully applied to the determination of lactate in blood diluted with PBS.

Polyethyleneimine (PEI), a cationic polymer, was used to immobilise lactate oxidase to the surface of a screen-printed carbon electrode, which was subsequently applied to the determination of lactate in peritoneal drain fluid samples [30]. PEI possesses a strong positive charge in aqueous solutions enabling electrostatic binding of the enzyme to the surface of the electrode.

Interferences such as ascorbic acid did not generate an electrocatalytic response. Shimomura et al. [31] detailed an alternative method for the novel fabrication of a lactate biosensor by coating a layer of lactate oxidase immobilised within mesoporous silica using a polymer matrix of denatured polyvinyl alcohol. The mesoporous silica encapsulates the lactate oxidase; this prevents the denaturing of the enzyme, followed by further entrapment within a polymer matrix of modified polyvinyl alcohol. This layer is then cross-linked and deposited onto the underlying Nafion layer.

Two strategies for the fabrication of a reagentless lactate dehydrogenase biosensor, used in a flow-injection system, are discussed by Prieto-Simón et al. [32]. The first strategy involves the use of a sol-gel matrix, which consists of tetraethyl orthosilicate (TEOS), water, ethanol and hydrochloric acid. The enzyme/cofactor and a number of binding agents are added to the mixture then deposited on the sensor surface. The second method involves the preparation of a mixture of polysulfone solution, graphite and a redox mediator. Both processes are fairly complex fabrication processes. The biosensor fabricated using the polysulfone-graphite composite displayed a high sensitivity of 80 mA/M, whereas the biosensor fabricated with the former method showed poorer performance.

### 2.5. Proteins

The determination of proteins is important in a variety of biomedical and food applications; consequently, simple, reliable and novel analytical approaches should be of considerable interest to clinical chemists and food analysts. Electrochemical immunosensors offer an attractive alternative to conventional methods, as they exploit the selective interaction of Ab/Ag interactions coupled with the high sensitivity afforded by the use of electrochemical techniques.

An immunosensor for the detection of C-reactive protein (CRP), a known marker of inflammation in human serum, has been developed by Kokkinos et al. [33]. This detector is based on a sandwich-type immunoassay which captures CRP at the sensor surface; the captured CRP reacts with streptavidin-conjugated PbS quantum dots. The quantification of the CRP is derived from the acidic dissolution of the quantum dots. The released Pb^+2^ is then determined by adsorptive stripping voltammetry at the surface of the electrode. Following optimisation, the biosensor was applied to the determination of CRP in spiked serum samples and validated with an ELISA kit. Recoveries of 96% and 106% were attained, with relative errors of less than 6%. In further work, Kokkinos et al. [34] have described a Bi-citrate screen-printed electrode (SPE) for the detection of DNA, specifically a C634R mutation of the RET gene. The analytical response was determined in a similar manner to the previously reported biosensor; an excellent LOD of 0.03 pmol·L^−1^ was achieved.

A screen-printed lab-on-a-membrane foldable device was developed for the duplex determination of biomolecules with metallic ions released from quantum dots [35]. This allowed for the simultaneous determination of both Pb^2+^ and Cd^2+^. Subsequently, this was applied to the determination of bovine casein (CN) and bovine immunoglobulin G (bIgG) in milk samples. Recovery values between 91%–108% and 92%–104% were determined in untreated samples respectively. In order to demonstrate its applicability to industry, the determination of goats’ milk adulteration with bovine milk was investigated. The CN assay was able to adulteration levels below 1% (v/v) whilst the bIgG assay was capable of determining adulteration levels as high as 50%.

Xu et al. [36] have also described an antibody based electrode for the analysis of immunoglobulin G however in this application the human IgG was analysed. The authors used of polyethylene glycol (PEG) to immobilize and capture the antibody onto the surface of a screen-printed carbon working electrode. The analyte and the carbon sphere/gold nanoparticle (CNS/AuNp) composite was subsequently also bound to the surface. The analytical response was derived from the differential pulse voltammetric detection of AuCl^4−^ following electro-oxidiation in 0.1 M HCl, the LOD achieved for human IgG was 9 pg·mL^−1^.

An immunoassay for the detection of two important tumour markers, carincoembryonic antigen (CEA) and alpha-fetoprotein (AFP) [37]. An eight electrode array consisting of six carbon screen-printed working electrodes was used for the analysis. Horseradish peroxide labelled antibody acts as the electrochemical probe which gives rise to the analytical response, which is measured chronoamperometrically at a potential of −0.25 V (vs. Ag/AgCl). The immunosensor was applied to determine both CEA and AFP in human serum samples. The levels of the human tumour markers compared favourably with a radioimmunoassay method.

Viswanathan et al. [38] have described the fabrication of a disposable electrochemical immunosensor to detect for carcinoembryonic antigen (CEA) in saliva and serum. Monoclonal anti-CEA antibodies were immobilized on a polyethyleneimine wrapped MWCNT screen-printed electrode. In addition, ferrocene liposomes were also dropcoated onto the surface of the sensor. This resulted in the generation of the analytical response which was proportional to amount of CEA bound at the surface of the electrode. With regards to the measurement of CEA in serum and saliva samples, according to the authors, the proposed immunosensor performs better than conventional ELISA kits.

## 3. Screen-Printed Carbon Electrodes for Vitamin Analysis

Vitamins are a complex group of compounds with a diverse range of chemical structures that give rise to interesting electrochemical properties. As most vitamins are naturally electroactive, or electroactive under modified conditions, their properties continue to be exploited using electrochemical techniques.

### 3.1. Vitamin C

Of the vitamins reviewed here, screen-printed sensors developed for the analysis of L-ascorbic acid (vitamin C) have received the most attention. Table 5 summarises the performance characteristics of a selection of screen-printed devices developed for the analysis of L-ascorbic acid over the past decade. Further details of fabrication and subsequent application are described in the following section.

#### 3.1.1. Mediated Electron Transfer

**Figure 4:**
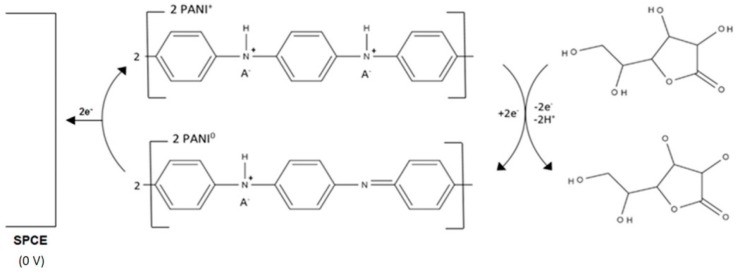
The reaction scheme of L-ascorbic acid at a polyaniline (PANI)-SPCE. Adapted from [40].

On their own, conducting polymers often lack the required mechanical [39] and conductive properties exhibited by carbon; however, their catalytic properties make them useful materials in the modification of SPCEs. Ambrosi et al. [40] used a simple drop-coating procedure to deposit nanoparticles of polyaniline (PANI) on to the surface of a SPCE; it was found that the addition of dodecylbenzene sulphonic acid (DBSA) during the synthesis of the conducting polymer resulted in the optimum catalytic response. The resulting nano-PANI-SPCE exhibited the widest linear range of all the sensors reported in this review (Table 5). The optimum operating pH was 6.8 and an applied potential of 0 V was feasible. This is the lowest reported operating potential for a conducting polymer-based L-ascorbic acid sensor; these conditions reduce the likelihood of other species present in pharmaceutical formulations producing an interfering response. The sequence of reactions involved in the operation of the PANI based sensor is summarised in Figure 4.

A PANI modified SPCE for the determination of L-ascorbic acid has also been reported by Milakin et al. [41]. The authors have similarly used a drop-coating procedure to deposit a mixture of aniline and ammonium persulfate in phosphate buffer; following deposition the aniline subsequently polymerises and the PANI binds to the carbon black surface. This PANI-SPC working electrode was used in conjunction with conventional counter and reference electrodes and successfully applied to the determination of L-ascorbic acid in a sample of grapefruit juice. The detection limit achieved with this device was the lowest reported over the past decade for SPCE based systems.

An alternative method for preparing PANI based sensors for L-ascorbic acid determination is using inkjet printing in conjunction with screen printing procedures [42]. In order to make a more environmentally friendly device, the authors chose a filter paper substrate for the three-electrode sensor. The response characteristics were inferior compared to the other PANI based sensors (Table 5); however, the authors suggest that the DBSA doping procedure reported by Ambrosi et al. [40] could improve their own device characteristics if adopted in the conducting polymer synthesis steps [42].

A different mediator namely quinoeimine was used for the development of an L-ascorbic acid sensor. In this case the fabrication procedure involved the electrochemical reduction of the in situ generated o-aminophenol diazonium salt [43]. The signal is generated by a two-step process. In the first step the L-ascorbic acid chemically reduces the *o-*quinoeimine form, which is followed by the electrochemical oxidation of the reduced form of the mediator. The comparison of performance characteristics places this sensor slightly below some of the mediator based SPCEs (Table 5) however, the selectivity achieved allowed a selection of fruits and juices to be analysed with little sample preparation [44].

#### 3.1.2. Unmediated Electron Transfer

**Figure 5:**
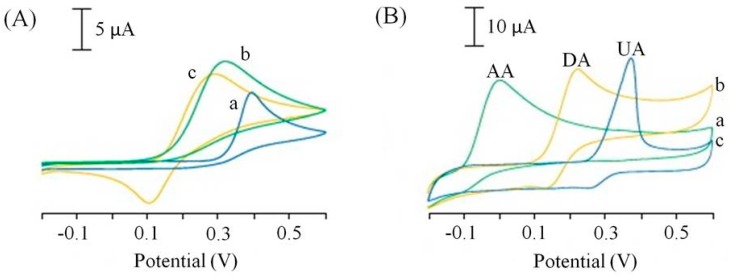
Cyclic voltammograms of 1.0 mM ascorbic acid (a), 1.0 mM dopamine (b), 1.0 mM uric acid at a screen printed electrode (**A**) and a screen-printed graphene electrode (SPGNE) (**B**). Supporting electrolyte: 0.1 M phosphate buffer saline (pH 7.0). Scan rate 50 mV/s. Adapted from [46].

All the publications reporting the use of SPCEs for the determination of L-ascorbic acid achieved a detection limits in the μM range, with the exception of a publication by Wonsawat [45] who reported a mM detection limit. This sensor used a commercially available screen-printed carbon working electrode without any reported surface modification [45]. However, a simple ink modification reported by Ping et al. [40] resulted in a thousand-fold increase in the limit of detection over Wonsawat [39]. The screen-printing ink was modified with a chemically reduced graphene oxide powder and accompanying ionic liquid (n-butylpyridinium hexafluorophosphate) [46]. In addition to the improved current response to L-ascorbic acid the screen-printed graphene electrode (SPGNE) also showed improved peak potential separation of L-ascorbic acid (AA) in the presence of two other important biological compounds, dopamine (DA) and uric acid (UA). These three compounds usually oxidise at similar potentials using traditional electrode materials, the improved peak separation and current responses are represented in the comparative cyclic voltammograms in Figure 5.

Another popular form of carbon for electrode modification is carbon nanotubes due to their unique electrochemical properties [47]. Moving further towards miniaturisation Crevillén et al. [48] fabricated a multi-walled carbon nanotube (MWCNT) SPCE coupled with a capillary electrophoresis microchip device using amperometric detection. The enhanced response characteristics achieved with a MWCNT modification are observable in the signal to noise ratios, LODs, and LOQs. The enhanced surface area from the nano-carbon structures was measured and a significant increase from 0.0075 cm^3^ for bare SPEs to 2.1 cm^3^ for MWCNT-SPCEs was observed. Although this device did not surpass the characteristics of some of the sensors in Table 5 the successful determination of multiple water-soluble vitamins, namely L-ascorbic acid, pyridoxine, and folic acid, was achieved. A publication by the same group used a similar sensor set-up to successfully determine a wide range of compounds important in food analysis; including polyphenols, vanilla flavours, and an isoflavones [49].

**Figure 6:**
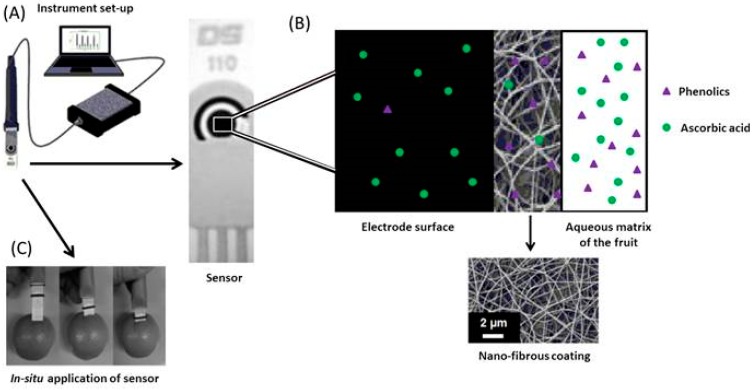
(**A**) Sensor connected to electronic hardware for data acquisition; (**B**) Schematic diagram of membrane role, with SEM image of nylon-6 coating; (**C**) Photograph demonstrating the in-situ analysis of ascorbic acid in fruit. Adapted from [50].

Membrane modifications such as that demonstrated by Fuenmayor et al. [50] often allow for in situ analysis of analytes. The nylon-6 nanofibrous membrane (N6-NFM) shown in Figure 6 was fabricated by electrospinning and subsequently applied to a SPCE for the direct determination of L-ascorbic acid in fruits. This membrane acts as a perm-selective barrier allowing vitamin C to pass to the underlying unmodified electrode whilst rejecting the interfering phenolic species. When compared to SPCE’s modified with mediators, this unmediated sensor possessed one of the widest linear ranges reported for SPCEs (Table 5, [40,41,42]). This advantage may be of practical use when performing analysis on foods and pharmaceuticals fortified with high concentrations of vitamin C.

### 3.2. Vitamin B

Vitamin B is the collective name for a large group of chemicals with an array of different structures. Table 6 displays the analytical characteristics and conditions for SPCE systems capable of determining the following compounds in the B vitamin group: vitamin B_2_ (riboflavin); vitamin B_6_ (pyridoxine); vitamin B_7_ (biotin); vitamin B_9_ (folic acid); and vitamin B_12_ (cyanocobalamin).

#### 3.2.1. Riboflavin

Kadara et al. [51] developed an unmodified SPCE for the determination of riboflavin; this electrode was successfully applied to the analysis of several food products. However, during these studies copper(II) was reported to have a significant effect on the signal response for riboflavin, which was initially seen as an interferent. In subsequent studies this interaction was exploited to significantly enhance riboflavin’s peak current response [52]. This signal enhancement from the addition of excess copper(II) was only observed in the presence of dissolved oxygen. Kadara et al. [52] propose this to be the result of a catalytic mechanism whereby the reduced form of riboflavin is reoxidised by the dissolved oxygen species. Another riboflavin sensor which also performed well in the presence of oxygen was developed by Riman et al. [53]; this sparked-bismuth SPCE was initially designed and applied to the voltammetric stripping analysis of Cd(II) and Pb(II) [54]. The SPCE was modified by a simple fabrication process involves a sparking procedure between the graphite SPCE and a bismuth wire [49]. This electrode exhibited superior analytical characteristics when applied to the analysis of riboflavin (Table 6), achieving a detection limit in the sub-nanomolar range using SWV [54].

#### 3.2.2. Pyridoxine

The two notable publications reporting the measurement of pyridoxine at SPCEs were performed at MWCNT-modified surfaces. Crevillén et al. [48] simply drop coated a suspension of CNTs onto the SPCE surface, and this working electrode was coupled with a Ag/AgCl wire reference electrode and a platinum wire counter electrode. This system was successfully applied to the determination of L-ascorbic acid (described in Section 3.1.2) and was also applied to a third vitamin, folic acid (Table 6); the simultaneous determination of all three vitamins was successfully achieved within a pharmaceutical formulation [48]. Although this system was applicable to a wide variety of analytes [49] it was unable to achieve the detection limit for pyridoxine reported by Brunetti et al. [55]. Additionally, this entirely screen-printed 3-electrode device was successfully applied to the analysis of food, drink, and pharmaceuticals.

#### 3.2.3. Biotin

**Figure 7:**
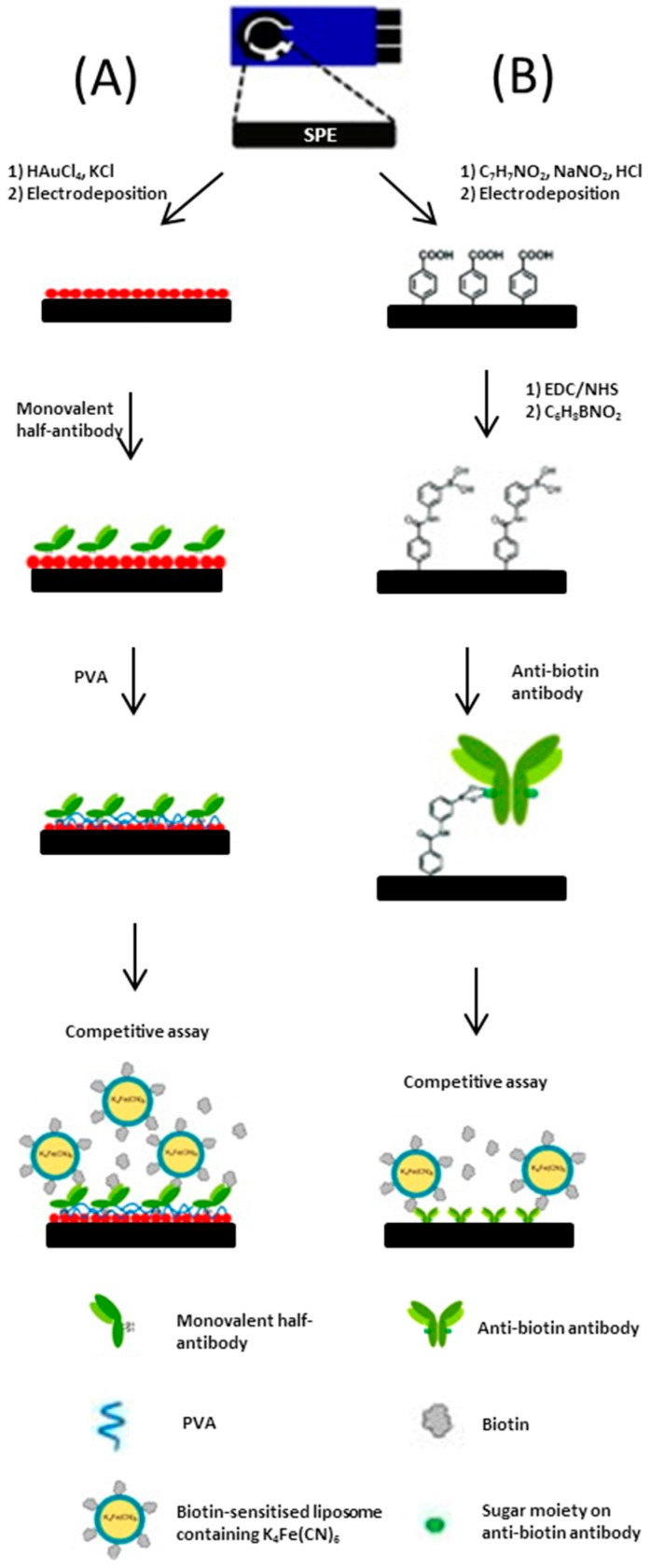
(**A**) Schematic representation of the method used to prepare a monovalent half-antibody/gold nanoparticles/screen printed graphene electrode electrochemical immunosensor and its mechanism of operation; (**B**) Schematic representation of the preparation of an antibody/anti-biotion antibody/screen printed graphene electrode electrochemical immunosensor and its mechanism of operation (drawing not to scale). Adapted from [57].

An assay developed by Ho et al. [56], utilises a SPCE as the base platform for a biosensor. The working electrode is constructed over four phases. In the first phase, a screen-printed carbon base layer is modified by the electrodeposition of a nano-structured gold network. Secondly, poly allylamine hydrochloride (PAH) is drop-coated onto the surface, which creates a 3D network for the addition of an anti-biotin antibody to be bound to. Following this the anti-biotin antibody/PAH/nano-Au/SPCE is immersed in a solution containing both biotin and biotin-tagged ferricyanide encapsulated liposomes with a short incubation period. In the fourth step, the addition of gold-nanoparticles was shown to significantly enhance electron transfer which resulted in increased sensitivity. The incorporation of a biological recognition element provides specificity for the voltammetric assay. The group reported further developments for a biotin immunosensor [57]; the two complex biosensors (depicted in Figure 7) use different binding techniques to enhance the orientation of antibodies on the SPCE surface. The most sensitive biosensor exploits the affinity of a sugar moiety on the anti-biotin antibody for the boronic acid-modified graphite surface.

Another competitive assay employing an electrochemical biosensor has been reported by Biscay et al. [58]; this sensor incorporates the biological recognition element onto the magnetic beads and a magnet is used for direct assembly over the working carbon electrode. The sensitivity of this sensor was improved in a subsequent publication with the use of a flow injection analysis (FIA) system [59]. This immunosensor-FIA system was successfully applied to the determination of biotin in two different pharmaceutical samples.

#### 3.2.4. Cyanocobalamin

Accumulation of vitamin B_12_ was achieved through the reduction of Co(III) to Co(I) which was pre-concentrated onto the surface of the electrode [60]. This was followed by the stripping step, whereby Co(I) was reoxidised to Co(II), which produced the analytical response. Initially the analytical signal was hampered by a large background current which was resolved by the addition of EDTA to the electrolyte solution. The final optimised analytical method allowed cyanocobalamin to be determined down to sub-nanomolar concentrations. A screen-printed graphite electrode, originally developed for the electrochemical determination of uranium [61], was recently applied to the determination vitamin B_12_.

## 4. Organophosphate (OP) Sensing

Very few research papers, related to the use of plain SPCEs, have been published in relation to the non-enzymatic determination of organophosphates (OPs) [62]. A rare exception by Li et al. [63], described the development of a photo-electrochemical assay using SPCEs with nano-sized titania surface modification with ultraviolet photocatalysis. By using differential pulse voltammetry, these non-selective sensors were able to measure 2 nM dichlofenthion in vegetables following extraction with a solvent. Enzyme-based biosensing for the detection of organophosphate and other pesticides has been the subject of considerable research effort since the 1990s and continued in the last 10 years (Table 7). Two enzymes form the basis of the majority of biosensing strategies developed over this time; organophosphate hydrolase (OPH) and acetylcholinesterase (AChE) [64]. However, butyrylcholinesterase (BChE) has occasionally been utilised as a direct analog to AChE. The acetylcholinesterase based system has been the most widely adopted, especially with respect to screen-printed electrodes; indeed, research featuring screen-printed electrodes for OP detection has almost entirely featured the AChE system in some form in the past 10 years. These biosensors have been repeatedly demonstrated as simple, rapid, and ultra-sensitive tools for pesticide analysis in environmental monitoring, food safety, and quality control. They have the potential to complement the classical analytical methods by simplifying or eliminating sample preparation and making field-testing easier and faster with a substantial decrease in cost per analysis [65]. The mode of operation of AChE-based biosensors is by the measurement of the reduction in analytical signal resulting from the inhibition of the enzyme in the presence of an OP. When AChE or BChE is immobilised on the working electrode surface, its interaction with the substrate (for example, with acetylthiocholine) produces an electro-active species (thiocholine) and its corresponding carboxylic acid [65]:

Acetylthiocholine + H_2_O + AChE → thiocholine (TCh) + acetic acid

The subsequent anodic oxidation of the thiocholine at the working electrode gives rise to a current that constitutes a quantitative measurement of the enzymatic activity:

2TCh (reduced) → TCh (oxidised) + 2H^+^ +2e^−^

The presence of pesticides in the sample inhibits enzymatic activity that leads to a drop in the current intensity, which is then measured. It should be noted that the sensitivity of these types of biosensors depends considerably on the chosen method of enzyme immobilization [62]. The adherence of the enzyme molecules onto electrode surfaces without denaturing the enzyme or blocking the active site has been a critical challenge for researchers. Various strategies have been used to immobilise AChE onto the electrode surface, including adsorption [66], entrapment [67,68] and cross-linking [69,70] amongst others. In an extensive study of immobilisation techniques Pohanka et al. [71] concluded that glutaraldehyde cross-linking was the preferred method and has proved to be a useful method for a range of electrode materials, such as gold [72], platinum [73] and carbon SPEs [74]. However, other strategies may be applicable depending on the composition of the sensor and surface chemistry.

In the past 10 years, there has been a high degree of diversity with respect to the composition and surface modification of screen-printed electrodes used in OP biosensors. Carbon remains the most common electrode material (Table 7) and has been used in the detection of OPs in sub-ppb concentrations [63,67,73,74], although gold [72] and platinum [75] have also been successfully used in the analysis of ppb levels of OPs.

From the published research it is unclear that any of electrode materials have an inherent electrochemical advantage for use in OP biosensors and the selection of electrode material appears to rely on their practicality, cost, and the experience of the research group involved. Gold and platinum SPEs have generally been used with surface modification for immobilising the enzyme only [71,72], whereas a wide and diverse range of electrode modifications have been made to the composition or the surface of SPCEs. These modifications have been to either entrap or cross-link the enzyme molecules to the sensor surface or commonly to improve the electrochemical properties of the working electrode.

A variety of nano-particles have been tested for surface modification, including those made of titania [63], gold/silver bimetallic [72], zinc oxide [76], manganese dioxide [71] and magnetic composite nanoparticles [77]. Multi-walled carbon nanotubes (MWCNT) [67] and single-walled carbon nanotubes (SWCNT) [78] have also been examined contributing to the detection of selected OPs at ppb levels. Gan et al. [79] successfully detected dimethoate at ppt levels with the use of magnetic composite nanoparticles in buffer and vegetable extracts; however other strategies have consistently resulted in the detection of low ppb concentrations of OPs. An example of his can be observed in the development of electric eel AChE-based biosensors by Chen et al. [67] who incorporated both MWCNT and tin oxide onto the surface of SPCEs. The analysis of simple vegetable extracts using these sensors with cyclic voltammetry resulted in a detection of 50 µg/L chlorpyrifos. The most commonly published modification for SPCEs in the past 10 years has been the inclusion of the electron mediator cobalt phthalocyanine (CoPC) within the carbon ink [70,78] or drop-coated onto the SPCE surface [74]. The addition of CoPC allows the electron transfer from the reduction of the substrate to the electrode at lower potentials thereby removing potential interferences. Practical advantages to the inclusion of CoPC within the electrode ink have been shown in the use of CoPC-modified SPCEs to create array-based systems to allow some identification as well as quantification of OPs in a substrate.

**Figure 8:**
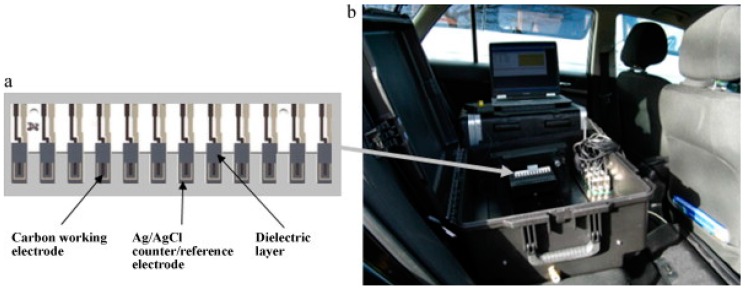
(**a**) Electrode array comprising 12 screen-printed carbon electrodes modified with cobalt phthalocyanine (CoPC) and an Ag/AgCl counter/reference electrode printed on an alumina substrate; (**b**) array in the prototype biosensor system operating in the field powered from a car battery via the lighter socket. Reproduced with permission [81].

The efficient electron-mediation combined with simple sensor designs allows the inexpensive manufacture of potentially commercially viable reproducible and sensitive sensor arrays. This is demonstrated in recent years by Alonso et al. [80], who used biosensors based on three separate AChE enzymes to differentiate chlorpyrifos and malaoxon in milk using chronoamperometry using an artificial neural network (ANN) to for signal interpretation. Additionally, Crew et al. [70] refined their previously developed AChE biosensor array systems for OP detection [81] to develop a portable prototype instrument for the analysis of five organophosphates using a wildtype and five modified *Drosophila melanogaster* AChE enzymes in an array format designed to be used with a standard 96-well plate (Figure 8a). The portable instrument was operated in the field using the power from a car battery via the lighter socket (Figure 8b). This prototype also used an ANN for signal interpretation and a simple three-minute inhibition step to allow rapid-analysis of food extracts or untreated environmental samples in the field. The inclusion of ANN analysis with flexible SPCE array formats for OP analysis belies the view that these sensors are not selective [82] and provides an optimistic future route for development for these biosensors.

## 5. Screen-Printed Sensors for Metal Ion Determination

Metal ions as pollutants are not chemically or biodegradable [82] and hence are ubiquitous and long lived contaminants. Their utilisation has led to planetary wide pollution [83,84,85] with the natural fluxes being greatly affected, impacting on a range of ecosystems [86,87,88]. Consequently, there is a pressing need for methods which are economic, precise and sensitive. Electrochemical techniques such as stripping voltammetry and the application of biosensors have been shown to be able to meet these demands. In terms of sensitivity and cost, few techniques approach the detection limits that can be gained by stripping voltammetry [89,90,91,92]. Portable, low-cost potentiostats can now readily be employed in the field or as a point-of-care technique.

In the past, stripping voltammetry has involved the use of Hg based working electrodes which, although possessing excellent electrochemical properties [93], they have suffered from perceived toxicity and disposal issues; this has resulted in a lack of market penetration. Alternative working electrode materials have been investigated such as carbon paste and glassy carbon [94,95]. Both these materials have been shown to exhibit excellent electrochemical properties, but necessitate a high level of skill on the part of the operator in order to obtained reliable reproducible. In contrast, screen-printing carbon based electrodes have been shown to be highly successful allowing for mass production with good precision. Recently, the application of SPCEs for the determination of metal ions has been reviewed by a number of authors [96,97,98,99,100,101,102]. Four approaches have been identified: Hg thin layer, Bi or Sb thin film and unmodified carbon electrodes using stripping voltammetry and to a lesser extent, biosensor based systems. These are described in the next four sections, and the performance characteristics of the sensing methods are summarized in Table 8, Table 9 and Table 10.

### 5.1. Unmodified Screen-Printed Carbon Electrodes

Unmodified SPCEs have been used as an alternative approach; depositing and accumulating the target metal ion as a metal film directly at the screen-printed carbon surface. Crew et al. [103] have used such an approach, for the determined Zn^2+^ in human sweat. The optimum supporting electrolyte was reported to be a pH 6.0, 0.1 M acetate buffer containing 0.1 M NaCl with a deposition potential of −1.6 V. Using a deposition time of 60 s a well-defined stripping peak at −1.2 V was reported with a linear response over the range 1 × 10^−8^ to 5 × 10^−6^ M. A coefficient of variation of 5.6% was obtained for six replicate measurements of a 2 × 10^−6^ M Zn^2+^ solution. The sweat from 10 volunteers was examined and Zn^2+^ concentrations of between 0.39 and 1.56 µg/mL were reported.

The determination of Pb^2+^ has been shown possible at a microband screen-printed carbon electrode (µBSPCE) [104]. The µBSPCE was fabricated by creating a 20 µm exposed cross-section of the printed layer of working electrode ink. Using a supporting electrolyte of 0.1 M pH 4.1 acetate 13 mM NaCl and an accumulation time of 1500 s under quiescent conditions a linear relationship was obtained from 50 µg/L to 1.7 mg/L with an associated detection limit of 2.3 ng/mL was reported by linear sweep anodic stripping voltammetry. The developed sensor was reported to be able to determine the concentration of lead leached from ceramic plates with solutions of acetic acid.

The determination of Au in human urine [105] by cathodic stripping voltammetry in 0.1 M KCl (pH 1.0) following open circuit accumulation has been reported at a poly-l-histidine modified SPCE. The stripping voltammetric determination of the accumulated Au was investigated by linear sweep (LSV), differential pulse (DPV), and square wave voltammetry (SWV). Detection limits of 6.0 µM, 1.7 µM and 4.0 µM were obtained respectively. SWV was found to be the most sensitive waveform, however, DPV was shown to obtain the lowest detection limit, and consequently was employed in subsequent studies.

Arduini et al. [106] have reported an amperometric based sensor able to determine levels Hg^2+^ as low as 1 ng/mL with response times of less than three minutes. The sensor was based on the interaction of Hg ions with the oxidation of thiocholine at a SPCE modified with a dispersion of carbon black N220. Significantly enhanced electrochemical activity was reported at SPCEs modified with the carbon black compared to unmodified SPCEs. Using a supporting electrolyte of 50 mM pH 7.4 phosphate buffer containing 0.1 M KCl, at an applied potential of +0.3 V (vs. Ag/AgCl) the amperometric response for both thiocholine and cysteine were found to be linear up to 1 × 10^−5^ M. The electrochemical oxidation of thiocholine and cysteine was reported to result from the oxidation of the thiol group to the corresponding disulphide. However, in the presence of Hg^2+^ ions the formation of a non-electroactive thiol-Hg complex was reported to be formed. The subsequent depletion in the amperometric response was found to be proportional to the concentration of Hg^2+^ ions present. This relationship was exploited by the authors as an analytical application for the determining trace Hg^2+^ levels in drinking water samples. It was reported possible to determine Hg^2+^ levels without the need for samples pre-treatment. For drinking water samples fortified with 5 × 10^−8^ M and 5 × 10^−9^ M of Hg^2+^, a signal decrease of 98% ± 2% and 14% ± 3% respectively was reported for a thiocholine at concentrations of 1 × 10^−7^ M. The effects for a number of other metal ions (Cu^2+^, Ag^+^, Pb^2+^, Fe^3+^, Fe^2+^, Ni^2+^, Mn^2+^ and As^3+^) were investigated at a concentration of 5 × 10^−6^ M using a thiocholine concentration of 1 × 10^−5^ M. Only the addition of Ag^+^ ions was reported to give any notable decrease in the amperometric response.

### 5.2. Mercury Modified Screen-Printed Carbon Electrodes

Thin-film mercury SPCEs (TFM-SPCEs) comprise a thin layer of Hg atoms adsorbed to the SPCE surface. This layer can be formed in several ways: via ex situ; deposition; plated before the analysis in a separate solution, or by in situ co-deposited with the target analyte by addition of a soluble Hg salt to the sample solution. Alternatively, an insoluble Hg salt can be mixed with the ink and printed as part of the working electrode, alleviating the need to for the addition of Hg by the end-user. Table 8 [107,108,109,110,111,112,113,114,115,116,117,118,119,120,121,122,123,124,125,126,127,128,129,130,131,132,133] gives a summary of these applications, highlighting the approaches employed and the performance characteristics of the devices. As can be seen from Table 8, TFM-SPCEs have been shown to be highly successful obtaining low limits of detection and precision. Nevertheless, even with the small amounts of Hg employed with these devices this approach still suffers from perceived issues of toxicity and disposal associated with Hg. In light of this perceived problem, two alternative strategies have been investigated are discussed in the next sections.

### 5.3. Bismuth Modified Screen-Printed Electrodes

A popular second alternative has focused on the use of the less toxic metal Bi [134,135]. This has been employed in a similar manner to that previously described for Hg, by in situ or ex situ deposition of soluble metal salt or alternating as an insoluble Bi salt, such as Bi_2_O_3_ [136,137,138,139,140,141,142,143] or BiPO_4_ [140] printed as part of the ink, which is then reduced to Bi° during the electrochemical deposition step. A number of reviews have been focused on the application of Bi modified electrodes [134,135]. Elements such as chromium [141], zinc [139], and lead [142] have been determined with Bi modified SPCEs.

Cobalt has been determined by cathodic adsorptive stripping voltammetry (CAdSV) at an ex situ Bi modified SPCEs [143]. In this reported Co was accumulated as its dimethylglyoxime (DMG) as complex. A series of soil extracts with varying Co concentrations were investigated. Results were compared to those obtained by inductively coupled plasma–mass spectrometry. Results showed the possibility of determining of levels as low as 0.1 µg/L.

Khairy et al. [144] have reported an in situ Bi film modified SPCE for the square wave stripping voltammetric determination of Cd^2+^ in artificial and diluted oral fluid. Investigations showed that unstable Bi film formation occurred if Bi concentrations were too large and a Bi concentration of 0.4 mg/L was reported to be optimum for the determination of 30 µg/L Cd^2+^. At pH conditions >3, magnitudes of the stripping peak current were reported to decrease, concluded to result from hydrolysis. Further investigations into the determination of Cd in oral fluid were reported to be effected by the adsorption of proteins and other components on the electrode surface. However, by adjusting the pH of the oral fluid samples to pH 1 the problem was alleviated. Investigations into the linear response of Cd^2+^ showed a linear range 10 to 80 µg/L using a deposition potential of 1.2 V. At Cd^2+^ concentrations above this, a decrease in the voltammetric response was found; concluded to result from saturation of the Bi layer. Based on a signal-to-noise ration of three (3 σ) a limit of detection of 2.9 µg/L was reported.

A number of more recent examples of the use of Bi-modified SPCEs for metal analysis are summarised in Table 9. However, a number of drawbacks of Bi modified SPCEs have been reported [145]. The use of Bi can result in narrowing of the potential range that can be readily used, an affect that can reportedly be aggravated by extremes of pH. The determination of elements such as Cu and Hg can be affected by these issues as they exhibit stripping peak potentials close to that of Bi. The addition of relatively large concentrations of Bi^3+^ to the sample solution required for in situ Bi film formation can lead to disruptions in the speciation of the target analytes. A further problem commonly reported with most metal thin-film based approaches is the possibility of peak splitting occurring if the optimum Bi concentrations are not employed. Nevertheless, a recent study of ex situ plated Bi electrodes for the stripping voltammetric determination Cd [142] showed Bi SPCEs were superior to Bi thin film glassy carbon electrodes which were shown to exhibited peak splitting.

An interesting combined stripping voltammetric-colorimetric assay has recently been reported by [146] for the determination of Pb^2+^, Cu^2+^ and Cd^2+.^ The SPCE was modified in situ with Bi, using an accumulation potential of −1.2 V for Cd^2+^ and Pb^2+^ and −0.6 V for Cu^2+^. The stripping step was undertaken in separate solution containing the metal indicator dye, xylenol orange. Metal ions formed during the electrochemical stripping step form a coloured complex with the xylenol orange allowing for their concentrations to be determined by UV spectroscopy at 575 nm for Pb^2+^ (acetate buffer) and Cu^2+^ (acetate buffer), 580 nm for Cd^2+^ (hexamethylenetetramine buffer), respectively. The possible Bi^3+^ ions formed in the stripping step were shown not to interfere with the spectroscopic determination of the determination of the target metal ions. Limits of detection were reported as 10, 10 and 100 nM for Cd^2+^, Pb^2+^ and Cu^2+^ respectively. The analysis of waste water samples showed good agreement with that obtained by inductively coupled plasma atomic emission spectroscopy (ICP-AES).

### 5.4. Alternative Metal Based Screen-Printed Electrodes

Alternatives to Hg and Bi have been investigated by Maczuga et al. [161]. In these investigations both electroplated and ink modified screen-printed Sb and Sn electrodes were investigated for the determination of both Cd^2+^ and Pb^2+^ by anodic stripping voltammetry. Antimony oxalate hydroxide, antimony oxide, and antimony tin oxide were investigated and a comparison of these modified SPCEs were made against both electroplated and ink modified Bi-SPCEs. The analytical performance of the Sb and Sn electrodes was reported to compare favourably with the bismuth-oxide modified bismuth electrodes. Detection limits were reported in the range 0.9–1.2 µg/L for Pb^2+^ and 1.8–3.5 µg/L for Cd^2+^ with a 240 s pre-concentration step. Mineral water samples spiked with Cd^2+^ and Pb^2+^ were reported to give percentage recoveries in the range 95% to 103%.

An automated anodic stripping voltammetric method for the determination of inorganic As has been reported by Punrat et al. [162]. The Au was electrochemically deposited onto the SPCE at a potential of −0.5 V vs. Ag/AgCl using a supporting electrolyte solution of 1 M hydrochloric acid. The linear range for the determination of arsenic (III) was reported as 1–100 μg/L, with a limit of detection in standard solutions of 0.03 μg/L using a deposition time of 120 s with a sample volume of 1 mL. The corresponding detection limit in real samples was reported to be 0.5 μg/L. It was reported possible to achieve speciation between arsenic (III) and arsenic (V) utilising deposition potentials of −0.5 V and −1.5 V for determination of arsenic (III) total arsenic concentrations, respectively. Similarly, good separation between the stripping voltammetric peak for As and that of the commonly interfering species Cu^2+^ was shown. Samples of rice field water and river water were investigated and good recoveries (99.5% to 104%) were reported for samples fortified with As^3+^ at 11.5 µg/L and 23.0 µg/L.

Ibuprofen derived gold nanoflowers/nanostructures (Ibu-AuPNFs/Ibu-AuNSs) have been synthesised by heating a mixture of ibuprofen and gold chloride (HAuCl_4_) at constant temperature [163]. The stability of the Ibu-AuNSs at the SPCE was improved by application of nafion. A linear response was reported for As(III) over the range of 0.1–1800 µg/L with corresponding detection limit of 0.018 µg/L. The sensor was reported to be highly reproducible with a coefficient of variation of 1.9% (*n* = 15) being reported. The selectivity of the device was reported to be very selective towards As(III) with no appreciable interference in the presence of various ions including Cu, Zn, Cd, Pb, Ni, Hg, Co, Ca, Na and K. The sensor was successfully employed for As(III) monitoring in various types of water samples.

The possibility of determining As^3+^ at a nanotube–Au nanoparticle-modified SPCE for the anodic stripping voltammetric determination of As^3+^ has been reported [164]. A mobile phone vibrating motor was attached to the SPCE to enhanced mass transfer. The peak current was reported to be linearly dependent on the As^3+^ concentration over the concentration range 10 to 550 µg/L. Detection and quantification limits were reported to be 0.5 and 1.5 µg/L, respectively when using a 120 s deposition time. It was reported that when using a 0.1 M HCl supporting electrolyte that Cu^2+^ did not interfere in the detection of As^3+^.

Cinti et al. [165] have reported the application of a carbon black (N220)-Au nanoparticle nanocomposite modified SPCE for the detection of As^3+^. SPCEs were modified by drop casting carbon black and Au nanoparticles onto the surface of the SPCEs. Using linear sweep anodic stripping voltammetry with a 0.1 M HCl +0.01% w/v ascorbic acid supporting electrolyte a detection limit of 0.4 µg/L was reported. No significant variation on the response for the determination of As^3+^ was reported in the presence of 100 µg/L of As^4+^. However, some effluence on the stripping response of As^3+^ was reported in the presence of Cu^2+^ at levels comparable with those occurring in tap water. No As^3+^ was reported to be detectable in the tap water sample investigated by the authors. Fortification of the sample with levels of 10 µg/L and 20 µg/L of As^3+^ gave average recovery of 99% ± 9% and 108% ± 4% for 10 ppb and 20 ppb As(III), respectively.

### 5.5. Biosensor Based Screen-Printed Electrodes

Certain metals readily interfere with enzyme activity; an effect which can be harnessed to develop biosensors for their determination [101,161,162,163]. The determination As^3+^ at an acetylcholinesterase modified SPCE has been reported [154]. It was shown possible to amperometically determine, at an applied potential of +0.6 V, the thiocholine iodide formed by the enzymatic reaction. Arsenic is known to inhibit acetylcholinesterase’s conversion of acetylthiocholine iodide to thiocholine iodide. Depletion of the amperometric response for thiocholine iodide can hence be used to quantify the concentration of As^3+^ present. Using this approach, a linear range up to 1 × 10^−7^ M As^3+^ could be obtained with a detection limit of 1.1 × 10^−8^ M. The developed biosensor was shown to be able to successfully determine 1.0 µM As^3+^ concentrations in tap water. Further investigations were made on a certified As^5+^ water sample. It was shown possible to determine As concentrations in this sample following the addition of sodium thiosulphate to reduce the acetylcholinesterase inert As^5+^ to As^3+^.

Guascito et al. [165] have utilised the widely used and commercially successful glucose oxidase enzyme system for the determination of a number of metal ions including: Hg^2+^, Ag^+^, Cu^2+^, Cd^2+^, Co^2+^ and Ni^2+^. Detection limits in the low µg/mL levels were reported with Ag^+^ detection limits in the µg/L region when as part of a flow injection system.

The interaction of arsenate with L-cysteine has been investigated for the development of a possible biosensor for As. L-cysteine reduces arsenate to arsenite and in process is oxidized to L-cystine. The reaction involves electron transfer which can be monitored amperometically [166]. By the immobilization of L-cysteine at the surface of SPCE, it was reported possible using this relationship to gain a detection limit of between 1.2 and 4.6 ng/mL for As. Interferences from oxidising agents such as nitrate were investigated, but no effects were reported at concentrations that are commonly found in drinking water.

The possibility of utilizing the inhibition of urease by Hg^2+^ was investigated at a SPCE modified with Au nano-particles [167]. The Au nano-particles were deposited electrochemically on the working electrode surface from a 0.1 mM solution of HAuCl_4_ in 0.5 M H_2_SO_4_. Au nano-particles were reported to enhance the sensitivity of the sensor. Using the modified SPCE a steady-state current was obtained for urea. Additions of Hg^2+^ were found to give a decrease in the urea current response proportional to concentration for Hg^2+^ added. Using this approach, a detection limit for Hg^2+^ of 5.6 × 10^−8^ M was reported. Using the developed biosensor, it was found possible to determine Hg^2+^ levels of 1.0 µM in fortified human plasma samples.

The metal-reducing bacterium, *Shewanella* sp. is involved in the cycling of several metals, such as, iron and manganese, as well as phosphates. Prasad et al. [168] have shown the possibility of using *Shewanella* sp. as the electron transfer material for electrochemical determination of arsenite, hydrogen peroxide, and nitrite. A *Shewanella* sp. CC-GIMA-1 bacterial suspension in 0.1 M, pH 7.4 phosphate buffered saline was drop-coated on the SPCE surface and allowed to settle under room temperature for 1 h. The approach was reported to be superior to the growing of bacterial biofilms on the SPCE surface. The presence of oxygen functional groups present on the electrode surface was considered to give a favorable environment for growth of the bacterial cells which utilised these oxygen groups as electron acceptors in their processes. The effects of arsenite (50–500 µM) hydrogen peroxide (50 µM–2.5 mM) and nitrite (100–500 µM) were studied by cyclic voltammetry at the modified SPCE. The addition of 500 µM arsenite resulted in a ca. 65% increase in the voltammetric reduction peak. Similar voltammetric behaviour was reported for additions of Fe^3+^.

Viguier et al. [169] have developed a self-assembled peptide nano-fibril modified screen-printed Au electrode (SPGE) as a biosensor for the determination of Cu^2+^. Four cysteine substituted forms of the octapeptide N-S-G-A-I-T-I-G (NS) were investigated as nano-fibrils: C-N-G-A-I-T-I-G (CN), N-C-G-A-I-T-I-G (NC) and C-S-G-A-I-T-I-G (CS). The SPGEs were modified by drop coating a 5 μL of a solution containing the nano-fibrils on the SPGE and incubating overnight at 4 °C. The SPGE was then washed with double-distilled water to remove unbound nano-fibrils. The functionalized SPGE was investigated by cyclic voltammetry, impedance spectroscopy, energy dispersive X-ray and atomic force microscopy. It was shown to be possible to accumulate Cu^2+^ ions on the modified SPGE at open circuit by immersing the biosensor into an aqueous solution of Cu^2+^ in 50 mM ammonium acetate (pH 6.8). Square wave voltammetry was then undertaken in 50 mM NaCl over the potential range −250 to 500 mV. Following the measurement step the biosensors were cleaned to remove any residual Cu^2+^ ions by applying a potential of 500 mV for 20 s in 0.1 M HClO_4_. Using a 2 minute accumulation time at open circuit, a linear response for Cu_2+_ over the concentration range 15 µM to 50 μM was recorded. Possible interferences resulting from Ca^2+^ and Mg^2+^ were commented upon but were not investigated in this report.

Niu et al. [170] have utilised a DNA modified SPGE biosensor for the trace determination of Hg^2+^, gaining a detection limit of 0.6 nM (120 ng/L). SPGE was modified by with a self-assembled mono layer (SAM) of the thiol-functionalized oligonucleotide, 5′-SH-(CH2)6-TTGCTCTCTCGTT-3′ (P1). In the presence of Hg^2+^ ions this can hybridise with a second ferrocene substituted oligonucleotide, 5′-TTCGTGTGTGCTT-ferrocene-3′ (P2) by forming thymine–Hg^2+^–thymine (T–Hg^2+^–T) complexes. However, in the absence of Hg^2+^, the two oligonucleotides cannot hybridize due to the T–T mismatched bases, and consequently P2 cannot be fixed to the electrode surface, so no electrochemical signal is produced. The developed biosensor showed a linear response for Hg^2+^ over the concentration range 10–0.001 µM was reported, and no interferences were reported for several metal ions including, Mg^2+^, Ba^2+^, Cu^2+^, Co^2+^, Fe^2+^, Na^+^ and K^+^ at 1 mM concentrations on the response gained for a 1 nM Hg^2+^ solution. Table 10 shows a summary of some of these applications.

## 6. Conclusions

In this review, examples have been described which illustrate the wide potential of electroanalytical (bio)sensors based on SPCEs.

The fabrication methods for biosensor construction have been discussed with particular emphasis on enzyme immobilization techniques. For the applications, involving glucose, glutamate, lactate and galactose, discussed at the beginning of the review, the most popular immobilization technique is the method of entrapment/encapsulation. The resulting reagentless devices have been successfully applied to a wide range of challenging matrices including blood, skin and food. An important advancement has been the miniaturisation of biosensors using simple low-cost convenient procedures in order to produce biosensors with dimensions in the micron region. It has been shown that these devices could be exploited for use in real-time toxicity testing studies which has potential applications in pharmaceutical drug development.

For the measurement of some important water-soluble vitamins, a selection of interesting surface modifications of carbon electrodes has been described. For example, successful electrocatalytic responses for ascorbate were obtained with a PANI-modified SPCE; the operating potential achieved with these devices were much lower than conventional devices without PANI. An interesting example for the measurement of biotin was described that involved the modification of an SPCE with an antibody which allowed an improvement in the selectivity; the immunosensor was used in a competitive assay format and concentrations down to 0.19 pg could be determined. This demonstrates an important approach which could be adapted for the measurement of other vitamins and possibly biomarkers.

Sensors for environmental pollution by organophosphate pesticides has continued to attract the attention of electroanalytical chemists. Measurement systems based on biosensors incorporating acetylcholinesterase have been the predominant approaches of choice. The signal is obtained when the substrate is enzymatically converted to an electroactive product. The decrease in the signal which occurs when the enzyme is inhibited by the organophosphate constitutes the analytical measurement. Several mediators have been investigated for lowering the over-potential for the enzyme product thiocholine, including CoPC and PEDOT, which gives similar operating potentials. The typical detection limits are in the low part per billion range and can be used for investigating natural waters.

Similar approaches to that described for OP’s have been described for some heavy metal ions; for example, arsenic has been determined by the inhibition of the acetylcholinesterase. In another approach, a biosensor based on urease has been applied to the determination of Hg^2+^ ions. This cation has also been determined using an interesting approach involving oligonucleotide hybridisation. These approaches hold promise for the measurement of other heavy metal ions of environmental concern. Stripping voltammetry, in conjunction with a variety of modified screen-printed carbon electrodes has continued to be of significant interest for the trace measurements of a range of heavy metals; samples containing sub-parts per billion of heavy metal ions have been readily analysed using screen-printed carbon electrodes modified with nanoparticles.

From the above discussions, it is clear that the prototype screen-printed sensors and biosensors described could potentially be further developed to produce commercial devices. The driver for commercialisation mainly dependents upon the size of the market for a particular applications area. For example, the glucose biosensor market continues to be the dominant sector for biosensor commercialisation, due to the growing issue of diabetes. The glucose biosensor market is projected to reach $31 billion by 2022 [173], accounting for 85% of the world market for biosensors. By comparison a smaller market where commercial biosensors seem to be making inroads, is in the area of sports science. For example, the measurement of lactate is important for athletes undergoing different training regimes in order to improve their performance. A device based on screen-printing technology has been commercialised by Imani et al. [174] which is worn on the chest during exercise in order to monitor lactate levels. The development of devices to for use in this area is likely to grow in the future due to the enormous interest from the public in attending athletic events such as the Olympic games. Other potential compounds that could be analysed and aid in improving athlete performance are heat-shock-proteins or uric acid [175].

Commercial screen-printed biosensors for other analytes such as glutamate and galactose have yet to be developed. However, Pinnacle Technology [176] and Sarissa Biomedical [177] currently sell devices capable of monitoring glutamate flux in the brains of rats, suggesting that there is a potential market for a device capable of analysing glutamate.

As far as we are aware, there are no reported commercial immunosensors based on screen-printed carbon electrodes, however, they offer an interesting opportunity to utilise the specificity of the antigen-antibody reactions in order to detect biomarkers of disease.

Screen-printed biosensors for the analysis of glucose in food and lactate in aqueous samples, are currently available to purchase from Gwent Electronic Materials, indicating their commercial appeal [178]. These are readily incorporated into a hand held instrument supplied by GEM for the monitoring of glucose in food is also commercially available.

In summary, we believe that it is readily feasible to commercialise screen-printed (bio)sensors where there is a sufficient sized market for a particular analyte and its subsequent application. For a manufacturing perspective, some of the obstacles to overcome are the difficulty in reproducible devices where multiple fabrication steps are required. In particular, complexities with regards to the deposition of the biological element on the surface of the biosensor, whilst retaining activity can present a challenge. As a result, simpler fabrication processes are likely to result in commercialisation. In our experience of fabricating prototype devices, the main drawbacks of obtaining reproducible devices lies in the immobilization. This suggests that integration of the biological components into the ink formulation is a possible way forward. We recently described the fabrication of a novel screen-printed glucose microbiosensor array using ultrafast pulsed laser ablation following the deposition of screen-printing ink containing the enzyme, glucose oxidase [179]. It should be feasible to adopt a similar strategy for the development of many other biosensors by incorporating appropriate enzymes into a suitable ink formulation.

## Figures and Tables

**Table 1:** Reports of screen-printed carbon electrodes incorporating glucose oxidase for glucose determination.

Immobilization Technique	Mediator	Assay Time (s)	Lower Linear Range (µM)	Upper Linear Range (µM)	Sensitivity	Applied Potential (mV)	Storage Stability (Weeks)	Reference
Crosslinking with glutaraldehyde & Nafion	Prussian Blue	N/A	20	700	N/A	200	90% activity after 6 months	[8]
Crosslinking with glutaraldehyde & BSA	Osmium-polyvinyl pyridine wired HRP	60	0	700	28.24 nA/μM/cm	0	90% activity after 15 months	[9]
Drop coating	Ferrocene	N/A	50	1000	2.12 µA/mM	−100	100% activity after 3 months	[10]
Use of SWCNT	PVI	5	500	800	32 μA/mM/cm	300	90% activity after 1 month	[11]
Use of SWCNT	Osmium bipyridine-complexed PVI	5	200	6000	16.4 μA/mM/cm	300	90% activity after 1 month	[12]
Immobilization on paper disk	Prussian Blue	N/A	250	2000	2.13 µA/mM	−300	72% activity after 45 days	[13]
Enzyme contained within water-based ink	CoPC	20	270	2000	16.4 nA/mM	400	N/A	[14]
Enzyme contained within water-based ink	CoPC	400 s	*Buffer:* 450	9000	*Buffer:* 26 nA/mM	400	N/A	[16]
			*Culture Medium:* 2000	13,000	*Culture Medium:* 13 nA/mM			
Enzyme contained within water-based ink	CoPC	30	0	2000	7 nA/mM	400	N/A	[17]
Enzyme entrapped by electro-polymerization of PEDOT	Prussian Blue	N/A	1000	10,000	2.67 μA/cm/mM	−100	82% activity after 1 month	[18]
Drop coating	Prussian Blue	5	12.5	2560	20.09 mA/M/cm^2^	−50	91% activity after 30 days	[19]

**SWCNT**: Single walled carbon nanotube. **PEDOT**: Poly(3,4-ethylenedioxythiophene. **HRP**: Horseradish peroxidase. **PVI:** Poly(1-vinylimidazole). **CoPC**: Cobalt phthalocyanine.

**Table 2:** Reports of screen-printed carbon electrodes for galactose determination.

Immobilization Technique	Mediator	Assay Time (s)	Lower Linear Range (µM)	Upper Linear Range (µM)	Sensitivity	Applied Potential (mV)	Storage Stability	Reference
Cellulose acetate	CoPC	10	100	25,000	7.00 µA/mM/cm	500	100% activity after two weeks	[20]
Cellulose acetate	CoPC	10	1980	9520	7.27 µA/mM/cm	500	N/A	[21]

CoPC: Cobalt pthalocyanine.

**Table 3:** Reports of screen-printed carbon electrodes for glutamate determination.

Immobilization Technique	Mediator	Assay Time (s)	Lower Linear Range (µM)	Upper Linear Range (µM)	Sensitivity	Applied Potential (mV)	Storage Stability	Reference
Entrapment with chitosan	Meldola’s Blue	2	12.5	150	0.44 nA/µM	100	N/A	[22]
Entrapment with chitosan & MWCNTs	Meldola’s Blue	20	7	105	0.39 nA/µM	100	100% after two weeks	[24]
Drop coated onto surface of CNTs	None	<5	0.01	10	0.72 ± 0.05 μA/μM	950	92% after 24 days	[25]

**MWCNT**: Multi walled carbon nanotube. **CNT**: Carbon nanotubes.

**Table 4:** Reports of screen-printed carbon electrodes for lactate determination.

Immobilization Technique	Mediator	Assay Time (s)	Lower Linear Range (µM)	Upper Linear Range (µM)	Sensitivity	Applied Potential (mV)	Storage Stability (Weeks)	Reference
Nafion	Variamine Blue	N/A	200	1000	0.46 nA/mM	200	N/A	[26]
Cellulose acetate	Meldola’s Blue	10	550	10,000	0.53 nA/mM	50	100% activity for 17 days	[27]
Enzyme contained within water-based ink	CoPC	100	1000	6000	3.63 nA/mM	400	N/A	[28]
Crosslinking with glutaraldehyde	Meldola’s Blue	5	100	10,000	3.46 μA cm/mM	0	N/A	[29]
Dropcoating onto a polyethyleneimine surface	Prussian Blue	5	200	800	3 µA/mM	0	N/A	[30]
Polyvinyl alcohol	CoPC	90	18.3	1500	4.54 μA/cm/mM	450	98% activity after 9 months	[31]
Polysulfone precipitation	Meldola’s Blue	30	1	125	80 mA/M	−100	75% activity after one week	[32]
CoPC: Cobalt pthalocyanine								

**Table 5:** Reports of screen-printed sensors for vitamin C (L-ascorbic acid).

Electrode Components	Supporting Electrolyte	Measurement Technique	Detection Limit (µM)	Linear Range (µM)	Sample/s	Modification Method	Reference
W: Nano-PANI SPCE R: Ag/AgCl C: Pt mesh	PBS pH 6.8	Amperometry 0 V	8.3	500–8000	Tablet pharmaceutical	Drop coating	[40]
W: PANI-SPCE R: Ag/AgCl C: GCE	0.05 M Phosphate buffer pH 7.0 & 0.5 M NaCl	Cyclic Voltammetry	0.1	1.00–80.00	Grapefruit juice	Oxidative chemical polymerisation	[41]
W: PANI-SPCE R: Carbon C: Carbon	0.1 M Acetate buffer pH 5.0	Chronoamperometry 0.4 V	30	30.00–270.00	None reported	Inkjet printed Paper based design	[42]
W: o-AP-SPCE R: Ag C: Carbon	0.1 M Phosphate buffer pH 7.2	Amperometry 0.2 V	0.86	2.00–20.00	Apple, Kiwi, Lemon, Orange, Pineapple, Strawberry, Tomato	Electrografted film	[43,44]
W: SPCE R: Ag/AgCl C: Carbon	0.1 M Phosphate buffer pH 2.0	DPV 0.0 V > −1.2 V	1360	1000–10,000	Orange juice	Unmodified	[45]
W: SPGNE R: Ag/AgCl C: Pt	0.1 M Phosphate buffer pH 7.0	DPV −0.2 V > +0.6 V Ep = −0.5 V	0.95	4.00–4500.00	Injection formula	Graphene ink formulation	[46]
W: MWCNT-SPCE R: Ag/AgCl wire C: Pt wire	0.01 M Phosphate buffer pH 7.0	Amperometry −1.2 V	11	50.00–400.00	Tablet pharmaceutical Capsule pharmaceutical	Drop coating	[48,49]
W: N6-NFM-SPCE R: Ag C: Graphite	Buffer citrate pH 4	Amperometry 0.35 V	Not reported	56.78–7381.33	Tangerine, Apple, Pear, Kiwi, Lemon, Strawberry	Electrospun membrane	[50]

**W**: Working Electrode. **R**: Reference Electrode. **C**: Counter Electrode. **PANI-SPCE**: Polyaniline screen-printed carbon electrode. ***o*-AP-SPCE**: *o*-Aminophenol film screen-printed carbon electrode. **SPGNE**: Screen-printed graphene electrode. **MWCNT-SPCE**: Multi-walled carbon nanotube screen-printed carbon electrode. **N6-NFM-SPCE:** Nylon 6 nano fibrous membrane screen-printed carbon electrode.

**Table 6:** Reports of screen-printed sensors for vitamin B compounds.

Analyte	Electrode Components	Supporting Electrolyte	Measurement Technique	Detection Limit	Linear Range	Sample/s	Reference
Vitamin B_2_ (Riboflavin)	W: Carbon R: Ag/AgCl C: Carbon	0.05 M Acetate-phosphate /KCl buffer pH 6.0	DPV −0.6 V > −0.2 V Ep = −0.42 V	2.39 µM	2.66–61.11 µM	Vitamin B premix, Dietetic milk powder, Corn flake cereal	[51]
Vitamin B_2_ (Riboflavin)	W: Carbon R: Ag/AgCl C: Carbon	0.10 M Acetate-phosphate /KCl buffer pH 8.0	LSV −0.1 V > 1.0 V Ep = −0.65 V	0.13 µM	0.016–0.399 µM	Variety of breakfast cereals	[52]
Vitamin B_2_ (Riboflavin)	W: Sparked Bi-SPCE R: Ag/AgCl KCl C: Pt wire	0.1 M Acetate buffer pH 4.5	SWV 0 V > +1.0 V Ep = +0.3 V	0.7 nM	0.001–0.01 µM	Tablet pharmaceutical	[53]
Vitamin B_6_ (Pyridoxine)	W: MWCNT-SPCE R: Ag/AgCl wire C: Pt wire	0.01 M Phosphate buffer pH 7.0	Amperometry +1.2 V	8.00 µM	25.00–300.00 µM	Tablet pharmaceutical Capsule pharmaceutical	[48,49]
Vitamin B_6_ (Pyridoxine)	W: MWCNT-SPCE R: Ag C: Carbon	Acetate buffer pH 5.0	DPV 0 V > +1.0 V Ep = +0.75 V	1.50 µM	2.00–72.00 µM	Tablet pharmaceutical Energy drink Cereal	[55]
Vitamin B_7_ (Biotin)	W: PAH/nanoAu/SPCE R: Ag/AgCl C: Pt	0.1M PBS pH 7.2	SWV +0.6 V > −0.3 V Ep = +0.2 V	8.30 nM	0.01 nM–0.01 M	None reported	[56]
Vitamin B_7_ (Biotin)	W: Ab/APBA/SPGrE R: Ag C: Carbon	Phosphate buffer pH 7.2	Amperometry −0.2 V	0.16 nM	0.1 nM–1.0 mM	None reported	[57]
Vitamin B_7_ (Biotin)	W: MonoAb/nanoAu/SPGnE R: Ag C: Carbon	Phosphate buffer pH 7.2	Amperometry −0.2 V	14.00 nM	1.0 nM–1.0 µM	None reported	[57]
Vitamin B_7_ (Biotin)	W: Carbon R: Ag C: Carbon	Phosphate buffer pH 7.2	Amperometry −0.2 V	Not reported	0.10–250.00 nM	None reported	[58]
Vitamin B_7_ (Biotin)	W: Carbon R: Ag C: Carbon	Phosphate buffer pH 7.2	Amperometry −0.2 V	Not reported	0.01–1.00 nM	Tablet pharmaceutical Liquid pharmaceutical	[59]
Vitamin B_9_ (Folic)	W: MWCNT-SPCE R: Ag/AgCl wire C: Pt wire	0.01 M Phosphate buffer pH 7.0	Amperometry +1.2 V	8.00 µM	50.00–400.00 µM	Tablet pharmaceutical Capsule pharmaceutical	[48,49]
Vitamin B_12_ (Cyanocobalomin)	W: SPGrE R: Ag/AgCl /3M KCl C: Pt	0.1 M Phosphate buffer, 0.1 M KCl, 10 mM/L EDTA pH 3	SWV −1.2 V > −0.3 V Ep = −0.73 V	0.07 nM	0.10–0.80 nM	Tablet pharmaceutical Liquid pharmaceutical	[60]

**Sparked Bi-SPCE:** Sparked bismuth screen-printed carbon electrode. **PAH/nanoAu/SPCE:** Poly allylamine hydrochloride nano-gold screen-printed carbon electrode. **Ab/APBA/SPGrE:** anti-biotin antibody-aminophenylboronic acid-screen-printed graphite electrode. **MonoAb/nanoAu/SPGnE:** Monovalent half-antibody-gold nanoparticles-screen-printed graphite electrode. **MWCNT-SPCE:** Multi-walled carbon nanotube screen-printed carbon electrode. **SPGrE:** Screen-printed graphite electrode. **W**: Working Electrode **R**: Reference Electrode **C**: Counter Electrode.

**Table 7:** Reports of screen-printed electrodes for organophosphate determination.

SPE Material	SPE Modification	Immobilization Method	Enzyme	Limit of Detection	Real Sample Analysis	Analytical Technique	Incubation Time	Reference
Gold	Cysteamine	Cross-linking	EE AChE	2 ppb paraoxon	Drinking water	CV	15	[67]
Carbon	MWCNT, SnO_2_, chitosan	Entrapment	EE AChE	0.05 µg/L chlorpyrifos	Vegetable extract	CV	14	[62]
Carbon	Carbon black, CoPC	Entrapment	BChE	18 nM paraoxon	Industrial waste water	Chronoamp	20	[69]
Carbon	MnO_2_	n/a	BChE	0.6 nM diazinon	n/a	Chronoamp	15	[71]
Carbon	Magnetic composite nano-particles, prussian blue	Entrapment	DmAChE	0.56 ng/L dimethoate	Vegetable extract	DPV	5	[72]
Carbon	n/a	Not declared	Not declared	n/a	Food extracts	CV/Chronoamp	n/a	[61]
Carbon	PEDOT, PSS	Entrapment	EE AChE	4 nM chlorpyrifos	n/a	Chronoamp	10	[63]
Carbon	SWCNT, CoPC	Cross-linking	EE AChE	5 ppb paraoxon, 2 ppb malaoxon	Water	Chronoamp	15	[73]
Gold	Glutathione, ZnO nanoparticles	Adsorption	EE AChE	10 ppb chlorpyrifos	n/a	CV	n/a	[70]
Carbon	Titania nanoparticles	n/a	n/a	2 nM dichlofenthion	Vegetable extract	DPV/Photoelec	n/a	[58]
Platinum	n/a	Entrapment	Human AChE	n/a	n/a	SWV/CV	5	[66]
Carbon	CoPC	Entrapment	DmAChE/PTE/EE AChE			Chronoamp	10	[74]
Carbon	CoPC	Cross-linking	DmAChE	n/a	Lake water	Chronoamp	10	[64]
Carbon	Ag/Pt bimetallic nanoparticles	Cross-linking	EE AChE/ChO	0.2 µM paraoxon/carbofuran	n/a	Chronoamp	10	[68]
Carbon	CoPC	Cross-linking	DmAChE	<1 nM pirimiphos/chlorpyrifos/ malaoxon/omethoate/dichlorvos	Food extracts, waste water, drinking water, river/lake water	Chronoamp	3	[65]

**Table 8:** Reports of screen-printed carbon electrodes for the determination of metal ions.

Analyte	Modifier	Medium Exchange	Accumulation Media	Measurement Technique	Linear Range	Detection Limit (Time)	Sample/s	Reference
Pb^2+^, Cd^2+^, Cu^2+^	Hg thin film	No	Sample acidified with HCl pH 2	SWASV, −1.1 V, 120 s	0–500 ng/mL in acidified seawater	Cd^2+^ 7.0 ng/mL, Pb^2+^ 0.31 ng/mL, Cu^2+^ 0.53 ng/mL	Seawater	[107]
Cr^6+^	Unmodified carbon	No	0.1 M H_2_SO_4_	LSCSV	100–1000 ng/mL	19 ng/mL	Canal water	[108]
Sb^3+^	Electrochemical generated silver nanoparticles	No	pH 2 Britton–Robinson buffer	DPASV, −0.6 V, (200 s)	9.90 × 10^−8^–9.09 × 10^−7^ M	6.79 × 10^−10^ M	Seawater, pharmaceutical preparations	[109]
Sb^3+^	Electrochemical generated gold nanoparticles	No	pH 2 Britton-Robinson buffer	DPASV, −0.55 V (200 s)	9.90 × 10^−8^–9.09 × 10^−7^ M	9.44 × 10^−10^ M	Seawater, pharmaceutical preparations	[110]
Sb^3+^	Mercury film	No	HCl 3 M	DPASV, −0.9 V (600 s)	0.99 × 10^−8^–8.26 × 10^−8^ M	1.27 × 10^−8^ M	Glucantime and seawater	[111]
U	4-Carboxyphenyl	No	Ammonium acetate	15 min	8.5 × 10^−10^–10^−7^ M	2 × 10^−9^ M	Estuarine water	[112]
Pb^2+^		No	0.1 M KCl	DPASV, −1.1 V (400 s)	10–60 μg/dL	2 μg/dL	-	[113]
Pb^2+^	Functionalized mesoporous silica	No	0.2 M HCl	SWASV, −1.2 V	1–30 ng/mL	0.1 ng/mL, 5 min accumulation, 120 s electrolysis	Drinking water, river water, groundwater	[114]
As^3+^	Platinum nanoparticle	No	1 M H_2_SO_4_	CV, −0.2 V to +1.3 V, 100 mV/s	1.6 × 10^−7^–1.3 × 10^−6^ M	5.68 ± 1.18 mg/L	Certificated water sample	[115]
Hg^2+^, Pb^2+^, Ni^2+^, Cd^2+^	PANI, or PANI-poly(DTDA)	No	0.1 M H_2_SO_4_; 0.5M HCl	DPASV, −0.4 V (120 s)	1 × 10^−9^–1 × 10^−6^ M	-	-	[116]
Cd^2+^	Hg modified microelectrode array formed by femtosecond laser ablation	No	acetate buffer 0.2 M, pH 4.5	SWASV	1–10 ng/mL	1.3 ng/mL (300 s)	River water	[117]
Cd^2+^	Ex-situ Hg plated thin film	No	acetate buffer 0.2 M pH 4.5	SWASV, −1.0 V	0.2–40 ng/mL	0.2 ng/mL, (60 s)	River water	[118]
Hg^2+^, Pb^2+^, Ni^2+^, Cd^2+^, Cu^2+^	Unmodified carbon	No	0.1 M NaCl, pH 1.35	DPASV, −1.4 V	-	-	Soil	[119]
Pb^2+^, Ni^2+^, Cd^2+^, Cu^2+^	Unmodified carbon	No	0.1 M NaCl, pH 1.35	DPASV, −1.4 V	-	-	Forensic soil analysis	[120]
Cd^2+^, Pb^2+^	Unmodified carbon	No	0.2 M acetic acid & 0.2 M sodium acetate	DPASV, −1.0 V	Cd^2+^ 2–100 µM, Pb^2+^ 5–100 µM	Cd^2+^ 500 nM, Pb^2+^ 800 nM (120 s)	Rainwater, flour, maize & seedlings	[121]
Pb^2+^, Cd^2+^	Thin-film Hg	No	0.6 M NaCl, pH 8	SWASV, −1.1 V	10–2000 ng/mL	Pb^2+^ 1.8 ng/mL, Cd^2+^ 2.9 ng/mL (120 s)	Seawater	[122]
Hg^2+^	PANI-methylene blue coated	No	0.5 M HCl	DPASV, −0.3 V	1 × 10^−8^−1 × 10^−5^ M	54.27 ng/mL (120 s)	Ultra-pure water	[123]
Hg^2+^	Electrochemically coated PANI-poly(DTDA)	No	0.5 M HCl	DPASV, −0.3 V	1 × 10^−8^–1 × 10^−5^ M	56 ng/mL (120 s)	-	[124]
Hg^2+^	poly(4-vinlylpyridine)	No	pH 4 acetate buffer + 2 M KCl	SWASV	100–1000 ppb	69.5 ppb	Skin-lightening cosmetics	[125]
Hg^2+^, Pb^2+^	Au film	Yes	0.05 M HCl	SWASV, −1.0 V	Hg^2+^ 2–16 ng/mL, Pb^2+^ 4–16 ng/mL	Hg^2+^ 1.5 ng/mL, Pb^2+^ 0.5 ng/mL, (120 s)	Drinking water	[126]
Cd^2+^, ^Cu2+^, Pb^2+^, Hg^2+^	Cd^2+^, Cu^2+^, Pb^2+^ by thin Hg film, Hg^2+^ Au screen-printed electrode	No	0.1 M HCl	SWASV, Hg^2+^ +0.2 V, Cd^2+^, Cu^2+^, Pb^2+^, −1.1 V	1 ng/mL–1 µg/mL for all	Hg^2+^ 0.9 ng/mL, (120 s), Cd^2+^, 1.0 ng/mL, Cu^2+^ 0.5 ng/mL, Pb^2+^ 0.3 ng/mL (300 s)	Dogfish muscle, Mussel tissue, Atlantic hake fillets	[127,128]
Cd^2+^, Cu^2+^, Pb^2+^	Injection modelled flow cell containing screen-printed sensor	No	Cu^2+^ 0.1 M HNO_3_, Cd^2+^ 0.1 M pH 9 ammonium citrate buffer, Pb^2+^ 0.1 M pH 9 glycine buffer	Cu^2+^ & Cd^2+^ DPASV, Pb^2+^ SWASV	Pb^2+^ 30–70 ng/mL, Cu^2+^ 9 ng/mL–26 ng/mL	Cu^2+^ 4.4 ng/mL (300 s), Pb^2+^ 5.9 ng/mL (500 s), Cd^2+^	Lake water, industrial waste water	[129]
Cd^2+^, Cu^2+^, Pb^2+^, Hg^2+^	Chitosan	No	0.1 M HCl/KCl	DPASV, −1.0 V	10–200 ng/mL	Pb^2+^ 3.4 ng/mL, Cu^2+^ 5 ng/mL, Cd^2+^ 5 ng/mL Hg^2+^ 2 ng/mL (30 s)	Tap water	[130]
Cd^2+^, Cu^2+^, Pb^2+^	Microchip capillary electrophoresis	No	MES buffer (pH 7.0, 25 mM)	−0.8 V	100–1000 µM	Pb^2+^ 1.74 µM, Cd^2+^ 0.73 µM, 0.13 µM	Green vegetable, Tomato and pine apple juices	[131]
Pb^2+^	Random micro-array formed by spraying screen-printed working with a commercial deodorant (200 mm for 12 s).	No	0.1 M HNO_3_	SWASV, −0.5 V	20–50 µM and 75–200 µM	9.5 µM	-	[132]
As^3+^	Au array for ASV, Pt array for direct oxidation, formed by spraying screen-printed working with a commercial deodorant (200 mm for 6 s).	No	1 M H_2_SO_4_	LSASV, −1.2 V	1–5 µM	4.8 × 10^-7^ M	-	[133]

**Table 9:** Reports of bismuth modified screen-printed carbon electrodes for the determination of metal ions.

Analyte	Modifier	Medium Exchange	Accumulation Media	Measurement Technique	Linear Range	Detection Limit (Time)	Sample/s	Reference
Zn^2+^, Cd^2+^ Pb^2+^	Chemically synthesized Bi nanoparticles	No	pH 4.5 0.1 M acetate buffer	SWASV, −1.4 V, flow cell & convective cell	-	0.52 ng/mL Zn^2+^, 0.45 ng/mL Cd^2+^, 0.41 ng/mL Pb^2+^, (120 s)	Waste water CRM, drinking water	[147]
Zn^2+^, Cd^2+^ Pb^2+^	bismuth oxide modified ink	No	0.1 M NaOAc solution containing 0.05 M HCl or 0.1 M HCl	SWASV, −1.2 V	Cd^2+^ 10–150 ng/mL, Pb^2+^ 10–150 ng/mL, Zn^2+^ 40–150 ng/mL	5, 10 and 30 ng/mL	River water	[148]
Cd^2+^	Microband ex-situ Bi plated	No	pH 4.5, acetate buffer 0.2M	SWASV, −1.0 V	5.6 ng/mL–45 ng/mL	1.3 ng/mL	River water (mining area)	[149]
Cd^2+^, Pb^2+^	Bismuth oxide modified ink	No	0.5 M ammonium acetate + 0.1 M HCl pH 4.6	Chrono-potentiometric	20–300 ng/mL	Pb^2+^ 8.0 ng/mL, Cd^2+^ 16 ng/mL	Soil, water	[150]
Zn^2+^, Pb^2+^	Ex-situ deposited bismuth	No	0.01 M KNO3	DPASV, −1.5 V, 60 s. stripping chrono-potentiometry	Up to: Zn^2+^ 250 ng/mL, Pb^2+^ 50 ng/mL, Cd^2+^ 600 ng/mL	Zn^2+^ 3.5 ng/mL Pb^2+^ 0.5 ng/mL, Cd^2+^ 3.9 ng/mL	Tap water (Barcelona)	[151]
Pb^2+^	Bi, 0.5% Nafion	No	10.0 mM acetate 50 mM KCl buffer + 500 mg/L Bi	SWASV, −1.0 V, 120 s	5 ng/mL–80 ng/mL	4 ng/mL	Leachates from cooking vessels	[152]
Zn^2+^, Cd^2+^ Pb^2+^	Dip coated hydrogel modified Bi doped ink	Yes	Volatile metal species generated at room temperature by the addition of sodium tetrahydroborate (III) to an acidified solution.	SWASV, −1.2 V	10–80 ng	1 ng (120 s)	Metal vapours	[153]
Zn^2+^, Cd^2+^ Pb^2+^	In situ plated Bi	No	1 M HCl	SIA-ASV, −1.4 V	2–100 ng/mL Pb^2+^ and Cd^2+^, 12–100 ng/mL Zn^2+^	0.2 ng/mL Pb^2+^, 0.8 ng/mL Cd^2+^, 11 ng/mL Zn^2+^	Herbs	[154]
Zn^2+^, Cd^2+^ Pb^2+^	In situ plated Bi	No	1 M HCl	SIA-ASV, −1.4 V	0–70 ng/mL Pb^2+^ and Cd^2+^, 75–200 ng/mL Zn^2+.^	0.89 ng/mL Pb^2+^, 0.69 ng/mL Cd^2+^	Drinking water	[155]
Cd^2+^ Pb^2+^	In situ plated Bi	No	0.2 M, pH 4.6 acetate buffer	SI-MSFA, −1.1 V	10 ng/mL–100 ng/mL	Cd^2+^ 1.4 ng/mL, Pb^2+^ 6.9 ng/mL	Water from a zinc mining draining pond	[156]
Zn^2+^, Cd^2+^ Pb^2+^	In situ plated Bi	No	0.1 M pH 4.5 acetate buffer, 10^−2^ M KCl	SWASV	10 ng/mL–100 ng/mL	Zn^2+^ 8.2 ng/mL, Cd^2+^ 3.6 ng/mL, Pb^2+^ 2.5 ng/mL	Tap water, waste water	[157]
Cd^2+^ Pb^2+^	Bismuth oxide modified ink	No	0.1 M, pH 4.5 acetate buffer	SWASV, −1.2 V	20 ng/mL–100 ng/mL	2.3 ng/mL Pb^2+^, 1.5 ng/mL Cd^2+^	River water	[158]
Pb^2+^	In-situ plated Bi Lab on a chip	No	0.1 M acetate buffer pH 4.5	SWASV, −1.2 V	2.5 ng/mL–100 ng/mL	1.0 ng/mL (120 s)	-	[159]
Pb^2+^	SPCE modified with filter paper containing electrolyte, Bi and internal standard (Zn) salts.	No	0.1 M pH 4.5 acetate buffer, containing Zn (60 ng/mL) as internal standard	SWASV, −1.4 V	10 ng/mL–100 ng/mL	2.0 ng/mL (120 s)	-	[160]

**Table 10:** Reports of screen-printed biosensors for the determination of metal ions.

Analyte	Modifier	Medium Exchange	Accumulation Media	Measurement Technique	Linear Range	Detection Limit (Time)	Sample/s	Reference
Cu^2+^, Cd^2+^ and Pb^2+^	Urease	sol-Gel	pH 7.0 0.02 mM phosphate buffer	Conductometric	0.1–10	-	-	[171]
Ni^2+^, Cu^2+^ and Cd^2+^	Acetylcholinesterase	-	-	Amperometric, TCNQ as mediator	Cu^2+^ 0.001–0.1	-	-	[172]
